# Effectiveness of a normative nutrition intervention (diet, physical activity and breastfeeding) on maternal nutrition and offspring growth: the Chilean maternal and infant nutrition cohort study (CHiMINCs)

**DOI:** 10.1186/s12884-015-0605-1

**Published:** 2015-08-18

**Authors:** Maria Luisa Garmendia, Camila Corvalan, Marcela Araya, Paola Casanello, Juan Pedro Kusanovic, Ricardo Uauy

**Affiliations:** Institute of Nutrition and Food Technology (INTA), University of Chile, Avenida El Líbano 5524, Macul, Santiago Chile; Department of Women and Newborn Health Promotion, Faculty of Medicine, University of Chile, Santiago, Chile; Department of Obstetrics and Gynecology, School of Medicine, Pontifical Catholic University of Chile, Santiago, Chile; Center for Research and Innovation in Maternal-Fetal Medicine (CIMAF), Department of Obstetrics and Gynecology, Sótero del Río Hospital, Santiago, Chile; Department of Pediatrics, School of Medicine, Pontifical Catholic University of Chile, Santiago, Chile; London School of Hygiene and Tropical Medicine, London, UK

## Abstract

**Background:**

Maternal obesity before and during pregnancy predicts maternal and infant risks of obesity and its associated metabolic conditions. Dietary and physical activity recommendations during pregnancy as well as weight monitoring are currently available in the Chilean primary health care system. However some of these recommendations are not updated and most of them are poorly implemented. We seek to assess the effectiveness of an intervention that enhances the implementation of updated nutrition health care standards (diet, physical activity, and breastfeeding promotion) during pregnancy on maternal weight gain and infant growth.

**Methods:**

*Design & Setting*: Cluster randomized controlled trial. The cluster units will be 12 primary health care centers from two counties (La Florida and Puente Alto) from the South-East Area of Santiago randomly allocated to: 1) enhanced nutrition health care standards (intervention group) or 2) routine care (control group).

*Participants:* Women seeking prenatal care before 15 weeks of gestation, residing within a catchment area of selected health centers, and who express that they are not planning to change residence will be invited to participate in the study. Pregnant women classified as high risk according to the Chilean norms (i.e age <16 or >40 years, multiple gestation, pre-gestational medical conditions, previous pregnancy-related issues) and/or underweight will be excluded.

*Intervention:* Pregnant women who attend intervened health care centers starting at their first prenatal visit will receive advice regarding optimal weight gain during pregnancy and diet and physical activity counseling-support. Pregnant women who attend control health clinics will receive routine antenatal care according to national guidelines. We plan to recruit 200 women in each health center. Assuming a 20 % loss to follow up, we expect to include 960 women per arm.

*Main outcome measures:* 1) Achievement of adequate weight gain based on IOM 2009 recommendations and adequate glycaemic control at 24-28 weeks of pregnancy according to ADA 2011, and 2) healthy infant growth during the first year of age based on WHO standards.

**Discussion:**

We expect that the intervention will benefit the participants in achieving adequate weight gain & metabolic control during pregnancy as well as adequate infant growth as a result of an increased impact of standard nutrition and health care practices. Gathered information should contribute to a better understanding of how to develop effective interventions to halt the maternal obesity epidemic and its associated co-morbidities in the Chilean population.

**Trial registration:**

Clinicaltrials.gov Identifier: NCT01916603

## Background

Obesity and related chronic diseases [cardiovascular diseases, cancer and diabetes (NCDs)] are currently the main causes of death and disability in adults in Chile and worldwide [1]. Although these conditions are observed in adulthood, there is now consistent evidence that NCDs originate in early life [first 1000 days (from -1 to + 2 years of life)] [2]. Several studies have shown that maternal body mass index (BMI) before conception and during pregnancy predict future risk of obesity and associated metabolic conditions for both mother and offspring [3–5]. Weight gain during the first two years of life is critical, not only to ensure newborn survival but also to define NCD susceptibility in later life [6–9]. This is a sensitive period in which nutrients and other environmental factors may condition gene expression and determine future health and NCD risks [10]. Thus, what we do or fail to do during the first 1000 days window provides a unique opportunity to decrease life-time NCDs risk [11]. Targeting this age group has an important potential impact given the current limitations in effectiveness of adult interventions [12], the wide coverage of maternal and infant programs and the lower cost of using existing resources. Unfortunately, maternal and infant programs have not adapted fast enough to meet the new challenges derived from the epidemiological and nutrition transitions, and obesity and NCDs continue to increase in most countries.

In Chile in recent years maternal and infant health and nutrition programs have been recognized as key to enhance human social capital [13]. However, from a nutrition perspective, these efforts have been insufficient to address the emergent NCDs. Excessive weight gain during pregnancy as well as childhood obesity have not been reduced but rather they have steadily increased particularly among the poor; presently, one out of three pregnant women is obese and 52 % of first-grade children report excess weight [14–16]. These trends are partly explained by implementation failures as well as delays in incorporate new knowledge in order to orient plan of action. This is particularly relevant in terms of nutrition recommendations and monitoring systems in place; these have not been fully updated to meet present international standards for nutrient intakes set by the World Health Organization (WHO) and the Food and Agriculture Organization (FAO) [17, 18]. There are now new recommendations (regarding the quantity and nutritional quality of the maternal diet and physical activity pattern during pregnancy) [19, 20] and monitoring goals (for both weight gain and glycaemic control) [21, 22] to support adequate fetal growth and maternal health during pregnancy and lactation [23]. Despite this, coverage of actions is often insufficient, i.e. specified actions are not always delivered on time, health personnel are not properly trained and/or beneficiaries do not attend scheduled visits. Several nutrition interventions during pregnancy focused on diet and physical activity have demonstrated their efficacy on reducing excessive maternal weight gain [24]; however, these studies are designed as high-intensity interventions in small sample sizes; thus limiting their applicability in clinical practice [25]. Further research is needed to confirm the effectiveness of low-intensity but high-coverage interventions on maternal and offspring outcomes and to assess the feasibility and ability of health care systems to deliver these interventions [26–28]. Thus, we propose a cluster randomized control trial (CRCT) [Chilean Maternal and Infant Nutrition Cohort Study (CHiMINCs)] to assess the impact of a low-intensity and high-coverage nutrition intervention by enhancing existing nutrition health care standards and practices at the primary health care level. We aim at increasing the proportion of women who achieve healthy weight gain and an adequate glycaemic control during pregnancy and the proportion of offspring who achieve a healthy growth during the first year of life.

## Methods/design

### Trial design

**Fig. 1:**
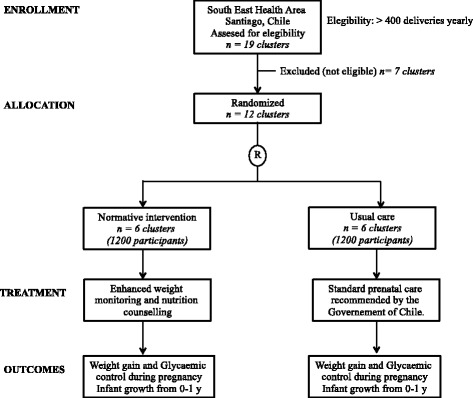
Flow chart for cluster randomized controlled trial comparing a normative nutrition intervention for pregnant women in public health care centers with usual care

This is a CRCT designed as a public health "program effectiveness" trial (Fig. 1). The proposed intervention will be delivered through the national health system under standard operating conditions, thus no extraordinary resource-intensive measures to secure uptake are unnecessary. The cluster units will be 12 primary health care centers (PHCCs) in two counties in the South-East of Santiago randomly allocated to: 1) enhanced implementation of nutrition health care standards (intervention group) or 2) routine care (control group). The desired outcomes will be the achievement of adequate weight gain at the end of the pregnancy, glycaemic control at 24-28 weeks of pregnancy and healthy infant growth at one year of age. Pregnant women from PHCCs randomized to the intervention will receive weight gain monitoring, diet and PA counseling-support and BF promotion from the first prenatal visit (<15 weeks) to 12 months postpartum. Pregnant women who attend control PHCCs will receive routine antenatal care according to national guidelines [29].

### Participants

Inclusion criteria for the clusters will be PHCCs from the two major urban counties of South-East Health Area of Santiago with > 400 deliveries yearly in order to achieve timely recruitment [La Florida (n = 5) and Puente Alto (n = 7)]; however, if we experience administrative or technical difficulties, we will consider replacing them with 4 smaller PHCCs. The unit of analyses will be mothers and their offspring. Women seeking care before 15 weeks of pregnancy, residing within catchment area of selected PHCCs and who are not planning to move in the next two years will be invited to participate in the study. Pregnant women classified during the first visit as high risk according to the norms of the Chilean Ministry of Health (Age <16 or >40 years; Medical conditions that exist before pregnancy, (hypertension, diabetes insulin dependent, kidney or heart chronic disease, drug abuse); Previous pregnancy-related issues (neonatal/fetal death, multiple abortions, birthweight < 2500 g or > 4500 g, premature delivery, birth defects, hospitalization due to hypertension or preeclampsia, previous uterine surgery, RH isoimmunisation; gestational diabetes mellitus); Pregnancy-related issues (multiple gestation, vaginal bleeding, pelvic mass**))** and/or underweight (BMI < 18.5 kg/m^2^) will be excluded. Other conditions that may affect either weight and/or glycaemic control during pregnancy or lactation and infant growth (i.e. severe depression, severe infections, injuries and trauma, congenital malformations) will not be considered as exclusion criteria. We have chosen to record these conditions and consider them in a secondary analysis.

### Recruitment and randomization

We will invite each of the 12 PHCCs to participate in the study using regular health service channels. We plan to randomly allocate half of the 12 PHCCs to the intervention arm of the study and half to the control group. Randomization will be done prior to recruitment of participants. All pregnant women who attend these PHCCs and fulfill enrollment criteria will be invited to participate in this study by the PHCCs midwives at their first visit; inclusion criteria will be checked and an informed consent will be sought from those who express an interest in the study. We expect to complete participant recruitment by the end of the first year of study (April 2015).

### Intervention

Overall, the CHiMINCs intervention is a low-intensity intervention designed to support the implementation of evidence-based guidelines by enhancing the uptake of existing programs (Table 1). The intervention for the intervened PHCCs has two main components: 1. Training to professionals: all midwives, dietitians and nurses will be trained on updated references, maternal weight gain assessment, use of charts, referral criteria to dietitian, dietary and physical activity recommendations, and how to communicate nutrition messages effectively. For this component several activities have been designed: one-day course, online refreshments (email and web page: http://www.chimincs.cl/), manual of procedures, technical support by the study coordinator, short brochure with nutrition messages, installation of a computer-assisted system for maternal weight monitoring based on IOM guidelines for weight gain during pregnancy in midwives´ consultation rooms. 2. Actions: *i.* Pregnant women at the first prenatal visit will be counseled regarding an optimal gestational weight gain range based on their pre-pregnancy nutritional status. At each routine midwife visit (mean prenatal visits in Chile = 6) weight gain will be assessed using the computer-assisted system for maternal monitoring and education and feedback about weight gain will be provided. Second, at each midwife visit pregnant women will receive advice about at least two of following nutrition messages (previously defined as more relevant by health professionals in a qualitative study): avoid the consumption of sugar-sweetened beverages (including fruit juice); restrict the consumption of white bread to two pieces/day; replace fatty meats (pork, veal, lamb) by lean meat (poultry, turkey) and fish; eat a variety of vegetables and fruits each day (at least 5 portions), in place of foods higher in fat and calories; antenatal breastfeeding promotion; invitation to physical activity classes. Third, midwife will refer pregnant women to dietitian according to defined criteria: gain more than 3 kg at the first trimester independently of their baseline nutritional status; gain more than 3, 2 and 1.5 kg at the second and third trimesters for normal, overweight and obese pregnant women at baseline, respectively. *ii.* A physical activity program for pregnant women of moderate-intensity exercise lasting 60 min and performed three times per week will be delivered at each PHCC supervised by licensed physical activity instructors. Each session will consist of 30 min of aerobic exercise of moderate-intensity (walking, dancing), 10 min of strength exercises and 10 min of stretching and elongating exercises.

**Table 1:** Nutrition intervention

1.	Training on weight monitoring and delivery of messages for health professionals:
	a. 1-day course for dietitians, midwifes and nurses: revision of references, maternal and offspring nutrition assessment, use of charts, referral criteria to dietitian, dietary recommendations, how to communicate nutrition messages effectively
	b. Online re-training (email and web page)
	c. Procedural manual
	d. Technical support by the study coordinator
	e. Short brochure with nutrition messages
2.	Installation of a computer-assisted system for maternal weight monitoring based on IOM (Institute Of Medicine) guidelines in midwives´ consultation rooms
3.	At each midwife visit (pregnant women):
	a. Assessment of maternal weight gain. Education and feedback about weight gain.
	b. Delivery of at least two nutrition messages: Avoid the consumption of sugar-sweetened beverages (including fruit juice); Restrict the consumption of white bread to two pieces/day; Replace fatty meats (pork, veal, lamb) by lean meat (poultry, turkey) and fish; Eat a variety of vegetables and fruits each day (at least 5 portions), in place of foods higher in fat and calories; Breastfeeding promotion; Invitation to physical activity classes.
	c. Timely referral to dietitian according to defined criteria
4.	Physical activity program for pregnant women of moderate-intensity exercise supervised by physical activity instructors lasting 60 min and performed three times per week.

### Routine care

Participants in the control group will receive standard prenatal care and nutrition counseling according to guidelines of Ministry of Health of Chile. All pregnant women are weighed at each prenatal visit but any further advice is given regarding adequate weight gain.

### Outcomes

Primary outcomes will be: 1) for mothers: a) adequate weight gain at the end of the pregnancy based on IOM 2009 [30]; b) adequate glycaemic control at 24-28 weeks of pregnancy according to ADA 2011 (having fasting plasma glucose (FPG) levels <92 mg/dl or 2-h values in the oral glucose tolerance test (OGTT) of <153 mg/dl) [22]; 2) for infants: adequate weight, length and BMI growth during the first year of life according to WHO 2006 [31] (Table 2). Secondary outcomes will be divided into: i) implementation outcomes (number of midwives and dietitians per PHCC, compliance to protocol by health personnel, time allocated to the actions of the interventions, etc.) and ii) participant compliance outcomes (attendance to dietitian's clinic, attendance to PA sessions, etc.).

**Table 2:** Outcomes of the intervention

Goal	Indicator
Healthy weight gain during Pregnancy^a^	Based on pre-conceptional nutritional status:
	Normal (BMI 18.5-24.9 kg/m^2^) =11.5-16.0 kg
	Overweight (BMI 25.0-29.9 kg/m^2^) = 7.0-11.5 kg
	Obese (BMI ≥30 kg/m^2^) = 5.0-9.0 kg
Adequate Glycaemic control during Pregnancy^b^	Fasting plasma glucose (FPG) levels <92 mg/dl or 2-h values in the 75-g oral glucose tolerance test (OGTT) of <153 mg/dl at 24-28 weeks of pregnancy
Healthy infant growth^c^from 0-1 y	Weight-for-age, height-for-age and BMI-for-age Z-scores based on WHO standards (between -2 and +2SD)

^a^Institute of Medicine (US) and National Research Council, Weight Gain During Pregnancy: Reexamining the Guidelines, 2009

^b^Diabetes Care, Vol. 34, Supplement 1, January 2011

^c^The Who Child Growth Standards, 2006

### Data collection

All data will be collected as part of routine health care activities. Study PHCCs have implemented electronic health records since 2009. Contact, sociodemographic information (eg. age, marital status, schooling, family structure, parity, etc.) and pre-pregnancy weight will be obtained from health records and by direct questioning at recruitment. All data related to pregnancy, delivery and offspring´s health are registered in a standardized manner in electronic health records; this system will allow evaluating all study outcomes. Electronic records will also provide with implementation and compliance data, which will be complemented with data obtained by the study coordinator by phone calls to a random sample of professionals and participants each two months. PA instructors will record PA attendance by participants at each session.

### Sample size

The sample size was estimated based on the hypothesized effect sizes using existing data, and the likely rates of study drop-out (20 %). Assuming that currently 50 % of Chilean women meet Institute of Medicine (IOM) weight gain recommendations [32], that we have six intervened and six control health centers, two-tail α p < 0.05; 80 % power and an intraclass correlation coefficient of 0.008 % [33] (based on cluster design of the study), we will need to recruit 200 women in each PHCC (n = 1200 per arm to obtain a final sample size of 960 per arm) to detect a 20 % difference in the achievement of IOM weight gain recommendations between intervened and control women.

### Statistical methods

Data analyses will be conducted by statisticians who will be blinded to the treatment allocation. Primary analyses will be carried out based on the allocation to each study group (“intention to treat”). Results will be presented as appropriate effect sizes with a measure of precision (95 % confidence intervals). All analyses will take into account the clustered design of the study (i.e. conduct multi-level regression analyses –hierarchical and longitudinal). Adjustment in the analyses will be done if there is baseline imbalance in covariates. Further exploratory analyses will be based on those participants who fully follow the protocol (“per-protocol analyses”).

### Safety monitoring

We will establish a data safety monitoring board with nutrition, obstetric, endocrinologist, and statistics experts. This group will be responsible for reviewing clinical trial data on an ongoing basis to ensure the safety of study subjects, as well as validity and integrity of the data**.**

### Ethical approval

The protocol for this study has been approved by the Institute of Nutrition and Food Technology of University of Chile, the Catholic University of Chile, and the South-Eastern Health Service Ethics committees. Potential participants will be provided with detailed information by the project field staff on the nature and relevance of the trial and the actions involved if they agree to participate. Potential participants will then be asked to sign a written consent form.

## Discussion

We expect that the intervention will benefit the participants in achieving adequate weight gain & metabolic control during pregnancy as well as adequate infant growth as a result of an increased impact of standard nutrition and health care practices. The results should be potentially extended to the rest of the primary health care system given the project´s "program effectiveness" approach and the participation of the Ministry of Health in the study design. Gathered information should contribute to a better understanding of how to develop effective interventions to halt the maternal obesity epidemic and its associated co-morbidities in the Chilean population.

## Abbreviations

NCDs: Non-communicable chronic diseases
PA: Physical activity
CHiMINCs: Chilean maternal and infant nutrition cohort study
PHCCs: Public health care centers
BMI: Body mass index
BF: Breastfeeding
